# Region and species dependent mechanical properties of adolescent and young adult brain tissue

**DOI:** 10.1038/s41598-017-13727-z

**Published:** 2017-10-23

**Authors:** David B. MacManus, Baptiste Pierrat, Jeremiah G. Murphy, Michael D. Gilchrist

**Affiliations:** 1 School of Mechanical & Materials Engineering, University College Dublin, Dublin, Ireland; 2 Ecole Nationale Supérieure des Mines de Saint-Etienne, CIS-EMSE, SAINBIOSE, F-42023 Saint Etienne, France; 3 INSERM, U1059, F-42000 Saint Etienne, France; 4 School of Mechanical & Manufacturing Engineering, Dublin City University, Dublin, Ireland

## Abstract

Traumatic brain injuries, the leading cause of death and disability in children and young adults, are the result of a rapid acceleration or impact of the head. In recent years, a global effort to better understand the biomechanics of TBI has been undertaken, with many laboratories creating detailed computational models of the head and brain. For these models to produce realistic results they require accurate regional constitutive data for brain tissue. However, there are large differences in the mechanical properties reported in the literature. These differences are likely due to experimental parameters such as specimen age, brain region, species, test protocols, and fiber direction which are often not reported. Furthermore, there is a dearth of reported viscoelastic properties for brain tissue at large-strain and high rates. Mouse, rat, and pig brains are impacted at 10/s to a strain of ~36% using a custom-built micro-indenter with a 125 μm radius. It is shown that the resultant mechanical properties are dependent on specimen-age, species, and region, under identical experimental parameters.

## Introduction

Internationally, traumatic brain injury (TBI) is the primary cause of death and disability in children and young adults, accounting for 15 deaths per 100,000 population per year in Europe, 30 in the U.S., and 120 in Colombia^1^. Traumatic brain injuries typically lead to neurocognitive deficits, psychological health issues, increased impulsivity, poor decision making, and impulsive-aggressive behaviour^2^. In Toronto, Canada, it was found that 45% of homeless men had a positive screening for TBI. Of these, 87% experienced their first TBI before they became homeless, and 73% before the age of 18^3^. The high occurrence of TBI has rendered it a significant public health and socioeconomic problem^2^. Furthermore, the pathophysiology and injury criteria of TBI, particularly mild TBI (mTBI), remains largely disputed^4^. While efforts to establish injury-criteria based on physical parameters such as head acceleration, velocity, and impact duration^5^, brain tissue strain^6,7^, and axonal strain and strain-rate^8,9^, have improved the diagnosis of TBI, they are susceptible to producing false-positives or false-negatives. The limitations of these injury criteria likely arise from the inherent heterogeneity of brain tissue which has been shown to change throughout an organism’s life^10–12^, is region-dependent^11–15^, rate-dependent^16–18^, and can be different depending on the chosen animal model^19,20^. However, combining the results reported in these individual studies to establish a holistic set of brain tissue properties is extremely arduous due to the differences in how the experiments were conducted. Furthermore, there is a dearth of accurate viscoelastic data for brain tissue at large strains and dynamic rates comparable to those sustained during head trauma. Here, we address these problems by investigating the effects of age, species, and region on brain tissue properties using identical experimental apparatus and conditions. It is shown that the mechanical properties of brain tissue are indeed dependent on age, species, region, and applied rate. More importantly, these properties can now be compared directly using statistical tests to determine if these differences are significant.

To achieve this, a custom built micro-indentation apparatus was developed to investigate the time-dependent mechanical properties of brain tissue at dynamic speeds and finite strain. A 125 μm radius indenter impacts regions of mouse, rat, and pig brains at 10/s strain rate to ~36% strain and is held for 1 s to allow for relaxation. This is the first time that the viscoelastic properties of brain tissue have been shown to be dependent on age, species, region, and rate for finite strain micro-indentation using the same experimental parameters, i.e. indenter radius, experimental temperature, data analysis, experimental apparatus.

## Theory

### Neo-Hookean hyperelastic model

Here, we assume brain tissue to be isotropic^21^ and quasi incompressible^22^ due to its high-water content^23^. A versatile and elegant model of hyperelasticity was proposed by Rivlin (1948), generalizing the corresponding linear theory in a natural way^24^. This so-called neo-Hookean model has the following strain-energy function:

1 $$W=\frac{\mu }{2}({I}_{1}-3),$$

where *μ* is the infinitesimal shear modulus and, if **C** is the right Cauchy-Green strain tensor, *I*_1_ = tr(**C**). The numerical difficulties associated with locally enforcing the incompressibility constraint in the displacement formulation of the finite element method  means that a slightly compressible version of the neo-Hookean model is often assumed when simulating soft tissue, with the following form typically used:

2 $${\rm{W}}=\frac{\mu }{2}({\bar{I}}_{1}-3)+\frac{1}{{D}_{1}}{(J-1)}^{2},$$

where $J\,\underline{\underline{{\rm{def}}}}\,\det ({\boldsymbol{C}}),\,{\bar{I}}_{1}\,\underline{\underline{{\rm{def}}}}\,{J}^{-2/3}{I}_{1}$ and ${\varkappa }$ is the infinitesimal bulk modulus, assumed to be 10,000 × *μ* for brain tissue. This value for bulk modulus assumes slight compressibility of the brain tissue with Poisson’s ratio of 0.49995^22^.

### Neo-Hookean-based viscoelastic framework

To accurately describe the viscoelastic response of neural tissue considering both the loading and relaxation phases, a quasi-linear viscoelastic (QLV) framework is used. The QLV theory assumes that the force *P*(*t*) exerted by an indenter on a viscoelastic material can be written as the convolution of a reduced relaxation function g(*t*) with an elastic force response function *P*^*e*^(*t*) as follows:

$$P(t)=\,{\int }_{-\infty }^{t}g(t-s)\frac{d{P}^{e}(s)}{ds}ds.$$

In this implementation of the QLV framework, *P*^*e*^*(t)* is the instantaneous elastic force determined from pre-computed neo-Hookean responses, over a range of shear moduli values, using the inverse finite element method^19,20^. Following Puso & Weiss (1998) it is assumed that the reduced relaxation function has the standard Prony series form^25,26^ i.e.,

$$g(t)={g}_{{\rm{\infty }}}+\sum _{i=1}^{N}{g}_{i}\exp (\frac{-t}{{\tau }_{i}}),\,$$

where *g*_*i*_ is the *i*^th^ relaxation modulus, τ_i_ is the *i*^th^ time constant, *t* is time, and *g*_*∞*_ is the long-term relaxation modulus. To ensure that the purely elastic response is recoverable from the viscoelastic model on letting τ_i_ → ∞, it will be required that:

5 $$g(0)=1,$$

and so therefore

6 $${g}_{\infty }=1-\sum _{i=1}^{N}{g}_{i}.\,$$

The elastic response should be recoverable from the viscoelastic model when *g*(*t*) = *constant* and therefore substitution of equation (4) into equation (3) gives the following viscoelastic response:

7 $$P(t)={g}_{\infty }({P}^{e}(t)-{P}^{e}(-\infty ))+\sum _{i=1}^{N}{g}_{i}\exp (\frac{-t}{{\tau }_{i}}){\int }_{-\infty }^{t}\exp (\frac{s}{{\tau }_{i}})\frac{d{P}^{e}(s)}{ds}ds$$

If *P*^*e*^(−∞) = 0, then the response has the form:

8 $$P(t)={g}_{{\rm{\infty }}}{P}^{e}(t)+\sum _{i=1}^{N}{g}_{i}{{H}}^{(i)}(t)$$

where

9 $${H}^{(i)}(t)\equiv \exp (\frac{-t}{{\tau }_{i}}){\int }_{-\infty }^{t}\exp (\frac{s}{{\tau }_{i}})\frac{d{P}^{e}(s)}{ds}ds$$

To numerically evaluate this function, use is made of the following recurrence relationship^26^:

$${{H}}^{(i)}(t+{\rm{\Delta }}t)=\exp (\,-\,\frac{{\rm{\Delta }}t}{{\tau }_{i}}){{H}}^{(i)}(t)+\frac{1-\exp (-\frac{{\rm{\Delta }}t}{{\tau }_{i}})}{(\frac{{\rm{\Delta }}t}{{\tau }_{i}})}({P}^{e}(t+{\rm{\Delta }}t)-{P}^{e}(t))$$

where *t* is time, Δ*t* is the time-step, *τ*_*i*_ are the *i*th time constants, and *P*^*e*^(*t*) is the elastic force.

## Materials and Methods

### Experimental Apparatus

**Figure 1:**
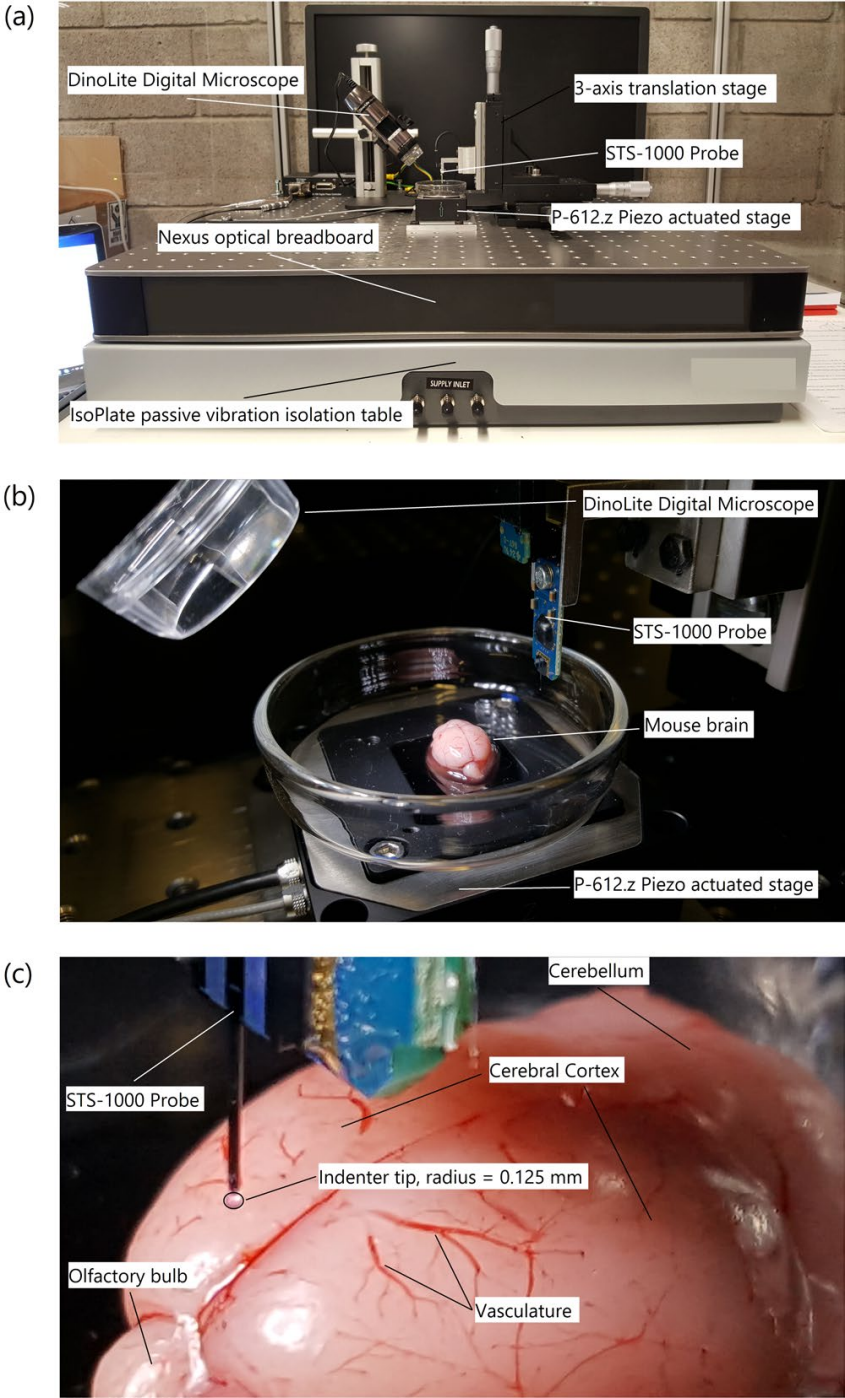
(**a**) Custom-built micro-indentation apparatus detailing the main components outlined above, (**b**) a close-up view of the piezo actuated stage and STS-1000 probe with mouse brain, and (**c**) close-up view of the mouse brain prior to indentation detailing gross anatomy of the brain and outlining the indenter tip.

A custom-built micro-indentation device was developed to investigate the mechanical properties of brain tissue at dynamic strain rates at localized length scales, (Fig. 1).

The custom-built device uses a Physik Instrumente P-612.Z piezo actuator stage to translate samples in the vertical direction (100 μm travel range) to impact the FemtoTools STS-1000 force-sensing probe. The piezo actuator uses closed-loop strain gauge control positioning with a closed-loop resolution of 1.5 nm, linearity error 0.2%, and repeatability of ±4 nm, calibrated in-house by Physik Instrumente using a Millitron Precision Gauge (calibration certificate supplied with order). The STS-1000 probes have a spherical ruby tip with a 125 μm radius, and a resolution of 0.05 μN at 10 Hz. The probes are connected via a proprietary cable to the FT-SC01 Force Acquisition System with a sampling rate of 10 kHz. The FTS-1000 probes are calibrated individually in-house by FemtoTools (calibration certificate supplied) and are provided with unique sensor gains (approx. 500 μN/V). The STS-1000 probes are fixed to a 3-axis translation stage with standard micrometers (engraved every 10 μm) for manual positioning of the probe and determination of contact point. A DinoLite AM7915MZTL (AnMo Electronics Corp., Taiwan) digital microscope is used to image the location of the indentation and ensure the area is void of vasculature. All the components mentioned above are fixed to a Nexus optical breadboard which is placed on an IsoPlate passive vibration isolation table (ThorLabs Inc. NJ, USA). The entire system is placed inside an aluminium-framed plexiglass enclosure (Machine Building Systems Ltd., Co. Westmeath, Ireland).

### Tissue preparation

#### Rodent Tissue

Mice and rats were euthanised by CO_2_ inhalation and collected on the day of testing from University College Dublin’s Biomedical Facility. All animals were euthanised by staff from the Biomedical Facility using authorised methods. All such staff are authorised by the Health Products Regulatory Authority, Ireland (HPRA). The specimens consisted of groups of 6, 10, and 12 W (W = week-old) mixed male and female mice and 20–25 W female rats. Mixed sex mice were used as it has been previously reported that gender has no effect on the dynamic compressive response of brain tissue^19^. Using mixed sex mice also allowed us to increase the sample size for each test. To perform the indentation experiments, the brains were removed from the animals by making a midline incision through the skin across the top of the head to gain access to the skull. A second midline incision, moving anteriorly from the occipital condyle, was made through the skull using a scalpel. Two lateral incisions were then made at an anterior and posterior point of the midline incision so that the bone could be removed and allow access to the brain. The brain was then separated from the spinal cord and removed from the skull. Brains were removed from the skull and indented individually, each brain remained *in situ* prior to testing. Proceeding removal from the skull, the brains were kept hydrated with Phosphate Buffer Saline (PBS) throughout the experiment. Considering the measurements were performed on post mortem tissue hydrating the samples with PBS was sufficient, forgoing the need to use artificial CSF to preserve tissue physiology. All tests were completed within 6 hours post-mortem to reduce the amount of proteolysis and necrosis that has been shown previously to reduce the stiffness of the tissue^27,28^.

#### Porcine Tissue

**Figure 2:**
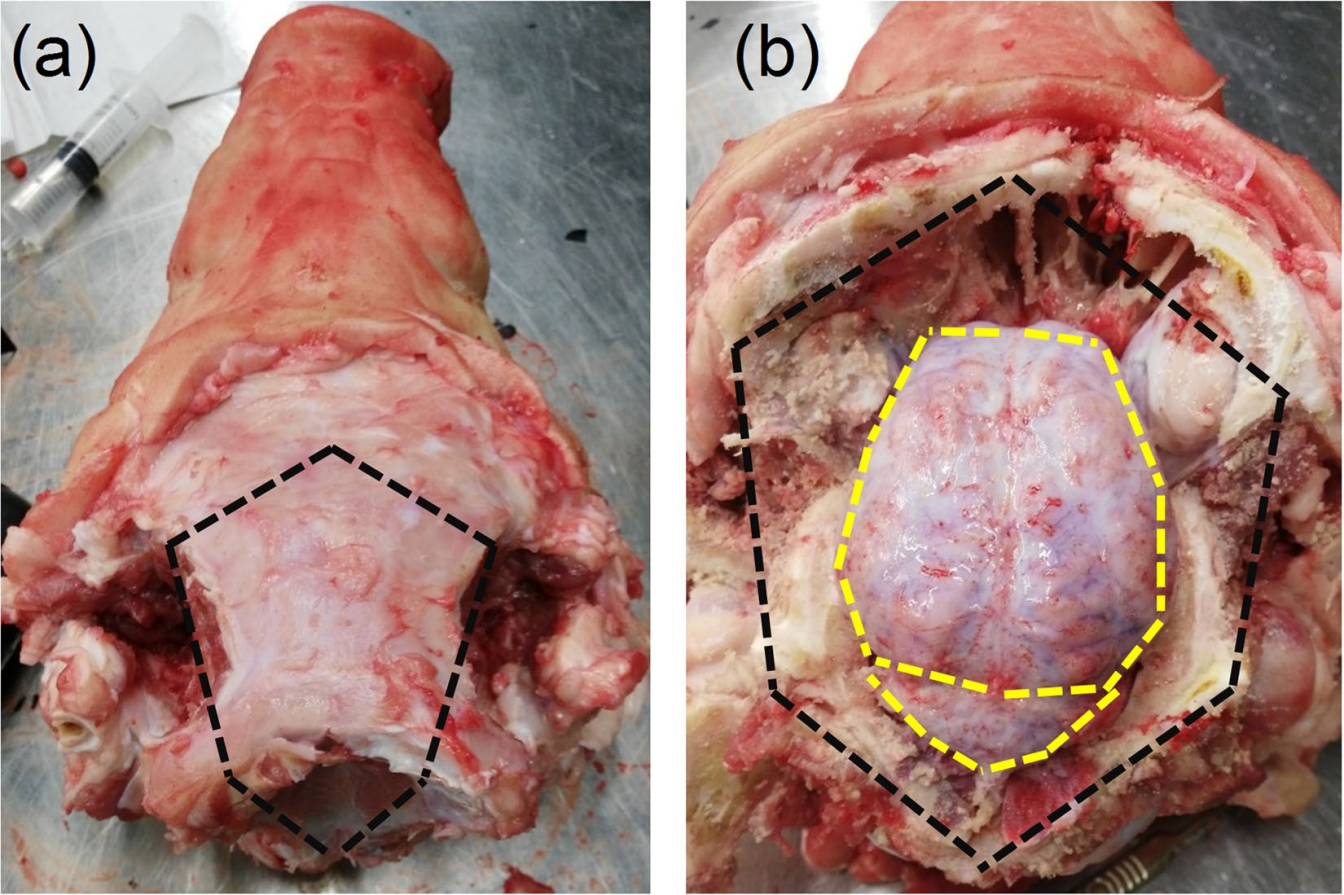
Removal of the pig brain. (**a**) Following resection of the scalp, the cranial bone was incised along the black dashed line taking care not to lacerate the meninges. (**b**) Once the cranial bone was removed, the meninges was incised along the yellow dashed line using a surgical scissors and taking care not to lacerate the brain tissue. The brain was then separated from the optic nerves, olfactory bulbs, and surrounding connective tissue. The brain is finally removed from the skull and placed in phosphate buffer saline prior to testing.

Porcine specimens were collected from a local slaughterhouse (Dawn Pork & Bacon, Waterford, Ireland) 4 hours before testing and transported to University College Dublin. The specimens consisted of four 22 W mixed sex pigs. Mixed sex pigs were used to increase the sample size on the given day of testing, and it has been shown previously that gender has no significant effect on the dynamic compressive response of brain tissue^19^. The scalp was removed using a scalpel exposing the cranial bone. Following the removal of the scalp, the cranial bone encasing the central nervous system was excised using an oscillating saw. Incisions using the oscillating saw were made in a pentagonal shape along the black dashed lines shown in Fig. 2a. Where possible, the incisions were made outside of the cranial cavity housing the brain to ensure the meninges and brain tissue were not damaged. Following removal of the skull, the meninges tissue was removed from the brain using surgical scissors incising along the yellow dashed line shown in Fig. 2b. Finally, following resection of connective and vascular tissue around the brain and separation from the spinal cord, the brain was removed from the skull and placed in PBS. Two of the brains were sectioned in the sagittal plane allowing the thalamus and corpus callosum to be indented. The remaining two brains were sectioned in the coronal plane at 37 mm from the prefrontal cortex allowing access to the corpus callosum and corona radiata. The prefrontal cortex was used for indentation experiments. The cerebellum from all four brains was sectioned along the sagittal plane and both hemispheres were used in the experiments. The brainstem was removed from all four brains; the medulla oblongata and pons regions were then separated. These sections were created due to the larger size of the pig brain, allowing the sections to fit into the petri dish that can accommodate the piezo stage. Two brains were extracted on each day of testing and were stored in PBS at 4 °C for up to a maximum of 2 hours prior to testing. All tests were completed within 8 hours post-mortem to minimize the amount of proteolysis and necrosis that has been shown previously to reduce the stiffness of the tissue^27,28^.

### Indentation protocol

Indentation tests were performed *in vitro* on the surface of the cerebral cortex (frontal lobe), cerebellum, pons and medulla of mouse, rat, and pig brains. Further indentations were performed on the corona radiata (coronal plane), thalamus (sagittal plane), and the corpus callosum (coronal and sagittal planes). The experimental groups and corresponding details are provided in Table 1. The full set of number of indentations per region per brain can be found in the Supplementary Material.

**Table 1:** Summary of experimental parameters, CX = cortex, CB = Cerebellum, MD = Medulla oblongata, PO = Pons, CR(C) = Corona radiata (coronal plane), TH(S) = Thalamus (sagittal plane), CC(C) = Corpus callosum (coronal plane), CC(S) = Corpus callosum (sagittal plane).

Animal	Specimen No.	Velocity (mm/s)	Strain	Indentation Depth (μm)	Indentation Locations (No. indentations)	Animal Age (weeks)
Mouse	3 (6 W), 5 (10 W), 2 (12 W)	1.65	0.36	57.7	CX; CB; MD; PO	6, 10, 12
Rat	4	1.65	0.36	57.7	CX; CB; MD; PO	22–25
Pig	4 (2 sagittal, 2 coronal)	1.65	0.36	57.7	CX; CB; MD; PO; CR(C); TH(S); CC(C); CC(S)	22

**Figure 3:**
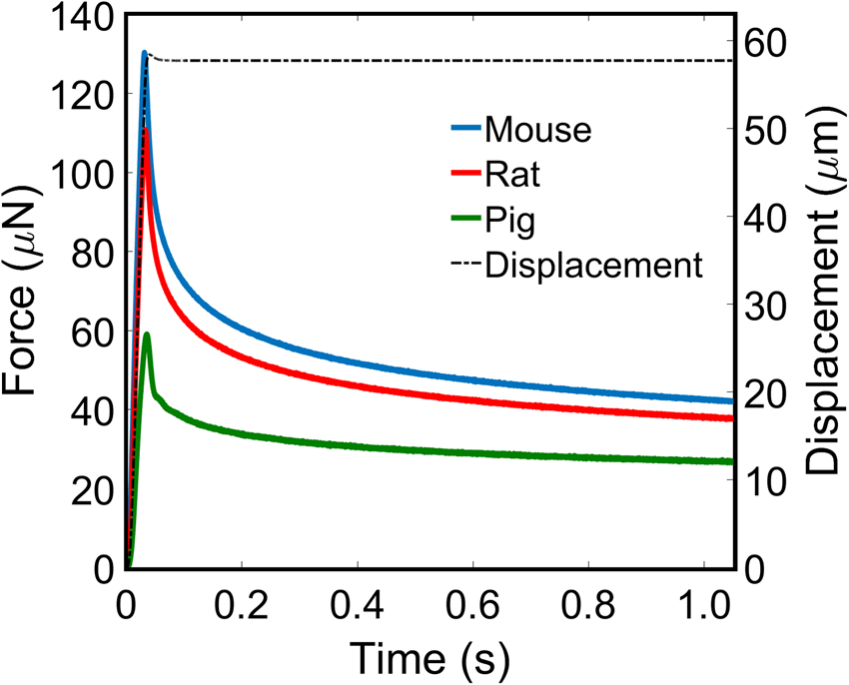
Mean force-time curves for the cortex of the mouse (blue), rat (red), and pig (green) brains. Velocity profile (black dash) of a 10/s indentation and 1 second relaxation. No filtering has been applied to the data.

Indentations were performed to 36% strain at 10/s as injury is likely to occur under these conditions^29,30^. The indentation depth required to achieve a strain of ~36% in the direction of the indentation was predetermined using finite element analysis to be 57.7 μm for a 125 μm radius spherical indenter. The strain measure used here is the maximum axial logarithmic strain below the indenter. Details of the finite element model are given below. To achieve a strain-rate of 10/s, indentations were performed at a velocity of 1.65 mm/s. The indenter was initially brought into contact with the brain by manually adjusting a 3-axis micrometer stage. If a force greater than 5 μN was recorded during establishment of the contact point the indenter was moved to a new location to perform the force measurement; no other preconditioning was performed on the sample. All tests were conducted in air at room temperature (~22 °C), continuously hydrated with PBS throughout experimentation, and discarded after each set of indentations. The neo-Hookean based quasi-linear viscoelastic model is fitted to the experimental data using the response surface method. No filtering is applied to the data prior to fitting of the mathematical model. A sample of mean force-time curves recorded from the cortex region of the mouse, rat, and pig brains are provided in Fig. 3.

### Optimization of viscoelastic parameters

The response surface method was used to determine the optimal values for the constitutive properties of brain tissue. The contact area is assumed to be locally smooth, and perpendicular to the axis of the indenter, due to the absence of gyri and sulci in the rodent brain, and the large surface area of the pig gyri compared to the indenter radius.

Axisymmetric analysis was used in the finite element model to take advantage of the indenter’s symmetry. The model consisted of a rigid indenter tip geometry, modelled as a 125 μm radius hemisphere consisting of 74 linear RAX2 line elements. The rigid indenter tip was initially in contact with the flat surface of a deformable cylinder, with a radius and height of 1250 μm, consisting of 3844 linear quadrilateral CAX4RH reduced integration, hybrid elements, representing a sample of brain tissue. A fixed boundary condition was applied to the opposing flat surface of the cylinder restricting motion in all directions, and a displacement boundary condition of 57.7 µm was applied to the indenter tip in the z-direction while motion of the indenter tip in other directions was restricted. Nonlinear geometry was used in this analysis due to the large deformation near the contact area. Using MATLAB, a script was created that performed the simulation for a range of shear moduli values between 0.1–10 kPa in 0.1 kPa steps for the neo-Hookean model and stored the corresponding force-time curves and shear moduli values in separate arrays. The length of the force-time curve array is extended to account for a 1 s hold time, initially treating the material as purely elastic (with no relaxation). Poisson’s ratio remained constant at 0.49995 for all simulations. A second MATLAB script used the hereditary integral approach given in equations (8–10) to convolve the numerically generated loading curves with the Prony series. Using the optimization algorithm, *fminunc*, and the sum of absolute differences method, the numerically generated curves were compared against the experimental data until the error was minimum and the material parameters were identified. It was also possible for the script to interpolate between the numerically derived curves when needed to obtain a better fit.

### Statistical analysis

ANOVA with a post-hoc test (Tukey-Kramer) was performed to determine if statistically significant differences exist between age groups, regions, species, and to test for anisotropy in the corpus callosum.

### Data Availability

All data generated or analysed during this study are included in this published article (and its Supplementary Material files).

## Results

### Age-dependent viscoelastic properties of brain tissue

Two likely sources of the disparities between the mechanical properties of brain tissue reported in the literature are the use of animals of different ages, and the assumption that brain tissue is mechanically homogeneous. Significant differences between brain regions has been previously shown for low-rate indentation^12,15,31^. Here, to elucidate the effects of age and brain region on large-strain dynamic properties of brain tissue, micro-indentation experiments were performed on 6 (n = 3), 10 (n = 5) and 12 (n = 2) week old mouse brains. The initial shear moduli derived from the quasi-linear viscoelastic model for each region and age group are presented in Table 2. Results from the ANOVA with post-hoc tests for significant differences between different regions are presented in Table 3. No significant differences were observed between the age groups in this study for the pons region. Significant differences were observed in the cortex between the age groups 6 and 10 W, and 6 and 12 W, in the cerebellum between 6 and 12 W and between 6 and 10 W for the medulla.

**Table 2:** Shear moduli for different regions of 6, 10, and 12-week-old mice.

Region	Age Group (_*μ*_, Mean ± SD, kPa)		
	6	10	12
Cerebellum	2.48 ± 0.39	2.81 ± 0.54	3.14 ± 1.08
Cortex	4.83 ± 1.01	6.75 ± 1.74	7.67 ± 0.48
Medulla	3.81 ± 0.86	4.65 ± 0.71	4.32 ± 0.57
Pons	5.66 ± 1.09	6.56 ± 1.69	6.51 ± 1.51

**Table 3:**
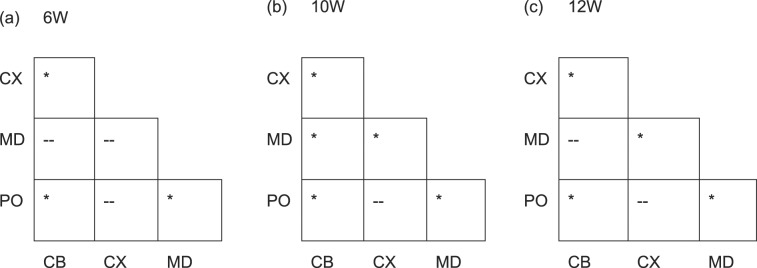
Post-hoc test results for significant difference between mouse brain regions. CX = cortex, CB = Cerebellum, MD = Medulla oblongata, PO = Pons. * = p < 0.01, -- = no significant difference.

### Effects of fiber direction on brain tissue mechanical properties

The degree of anisotropy in white matter tissue remains controversial^21,32^. Few studies have attempted to address this issue due to the size of the microscopic fibers and difficulty in establishing their true direction without the use of expensive equipment and complex analysis such as MRI and diffuse tensor imaging (DTI)^21^. Studies which have been conducted on the anisotropic property of brain tissue have had conflicting results^11,16,21^.

**Figure 4:**
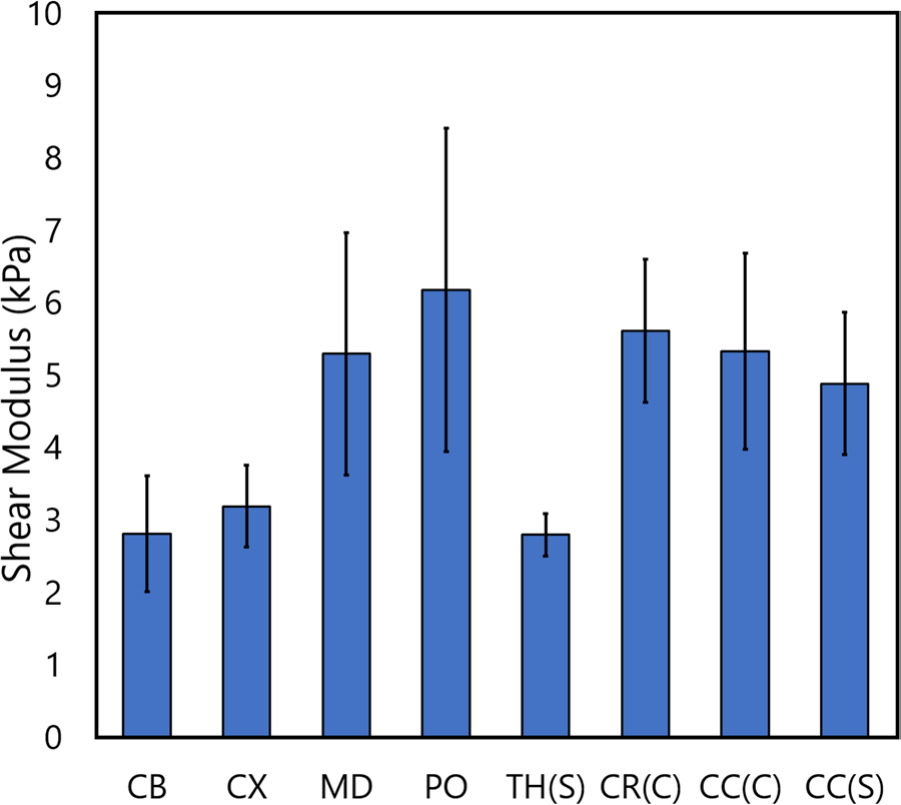
Mean ± standard deviation of the shear moduli values from regions of the pig brain. Statistical differences between these regions are outlined in Table 4.

To add to this body of evidence, we performed indentations on the highly-aligned corpus callosum region in both the coronal and sagittal planes of the pig brain, using results from Budday *et al*. as a guide for fiber direction^21^. Indentations were also performed on the more randomly distributed coronal radiata. It is clear from Table 4 that there is no significant difference between shear moduli values (Fig. 4) for either of the directions tested for the corpus callosum, which is arguably one of the most highly-aligned brain regions. Furthermore, no significant difference exists between either corpus callosum direction or the corona radiata. Also, no significant differences were found to exist between any white matter regions (medulla, pons, corona radiata, and corpus callosum), or between any grey matter regions (cortex, cerebellum, thalamus), which is contrary to the results reported here for the mouse and rat brains and results previously reported by Elkin *et al*.^15^. Significant differences were found to exist, however, between an average of all grey matter regions and an average of all white matter regions consistent with several studies in the literature^16,31,33–35^. The underlying cause of this phenomenon is unclear; however, we hypothesise that it is likely due to the age of the specimens used in this study. It is possible that significant differences within white matter groups and grey matter groups may occur with older pigs such as those observed by Elkin *et al*.^15^.

**Table 4:**
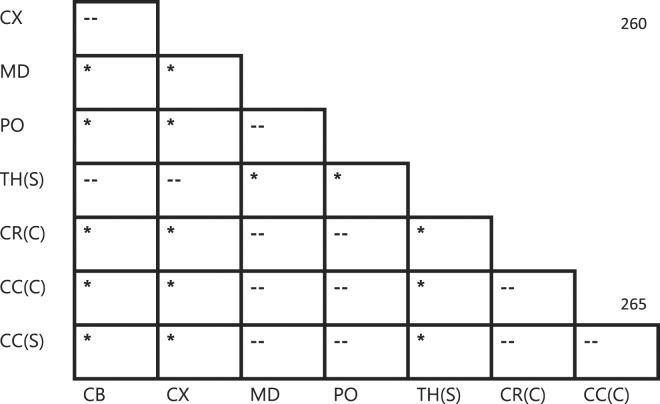
Statistically significant differences between porcine brain regions. CX = Cortex, CB = Cerebellum, MD = Medulla oblongata, PO = Pons, CR(C) = Corona radiata (coronal plane), TH(S) = Thalamus (sagittal plane), CC(C) = Corpus callosum (coronal plane), CC(S) = Corpus callosum (sagittal plane). * = p < 0.01, -- = no significant difference.

### Species-dependent viscoelastic properties of brain tissue

Micro-indentation data from adult animals are compared to investigate if significant differences exist for the cortex, cerebellum, medulla, and pons across species. All animals used in the statistical comparisons are assumed to be adults or adolescent. While there are some scientific data supporting the designation of adulthood for 10 W mice^36^ and the 20–25 W rats^37^, there is a lack of scientific evidence detailing the onset of adulthood in pigs. However, for this study we considered sexually mature pigs to be adolescent, as is the case with the 22 W pigs used here^38^.

**Figure 5:**
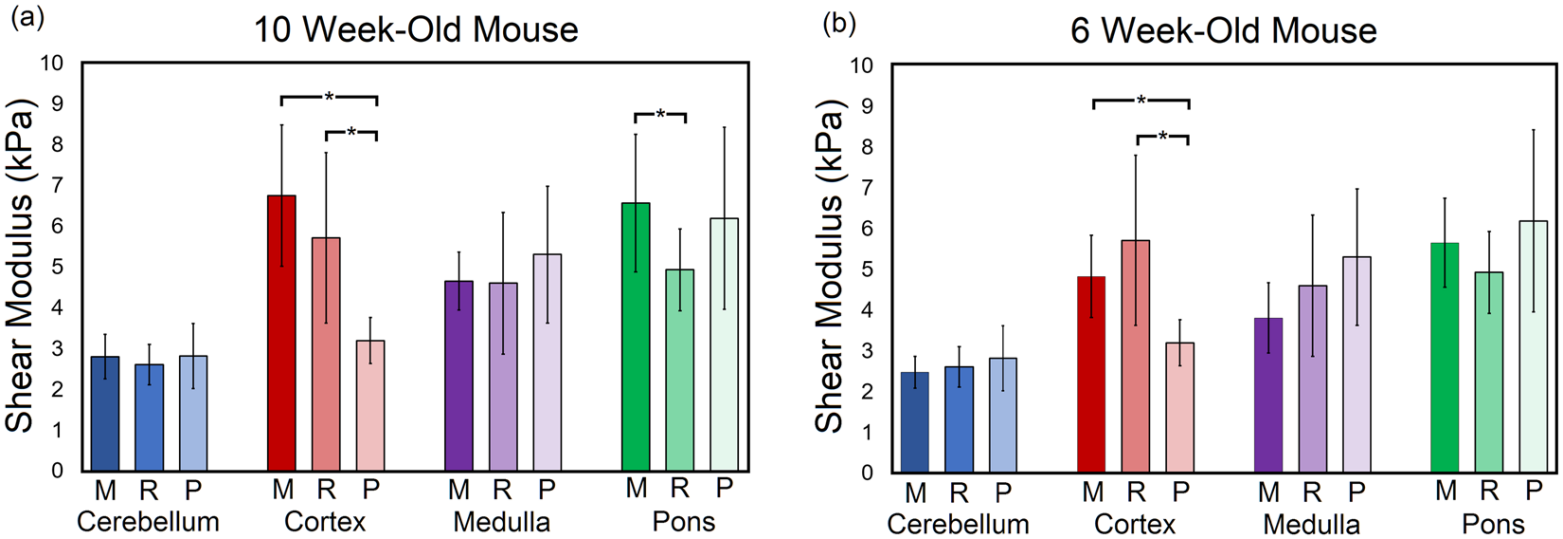
Mean ± standard deviation of the shear modulus values across species including (**a**) a 10-week-old (10 W) mouse model and (**b**) a 6 week-old (6 W) mouse model. Both (**a** and **b**) have a 20–25-week-old rat model and a 22 week-old pig model. ANOVA with a post-hoc Tukey test was performed for each region across the three species to determine whether significant differences existed for the material properties of brain tissue across species. *Denotes a significant difference, M = mouse, R = Rat, P = Pig. Full list of viscoelastic parameters for all groups are provided in the supplementary material.

ANOVA with post-hoc test for significance was performed to compare the shear modulus of the cortex, cerebellum, medulla, and pons regions between the mouse, rat, and pig brains. Figure 5 presents the results from the statistical analysis and the mean ± standard deviation of the shear modulus determined from the neo-Hookean based viscoelastic model. No significant differences were found between the cerebellum and medulla for all species, between the cortex region for mouse and rat tissue, or the pons for rat and pig, and mouse and pig tissue. Significant differences were observed, however, between the mouse and pig cortex, rat and pig cortex, and the mouse and rat pons. Substituting the 10 W (adult) mouse data with the 6 W (adolescent) mouse data, the mouse cortex remains significantly different from the pig cortex; however, a significant difference is no longer found between the mouse and rat pons region. No significant differences were observed for the cerebellum and medulla. To further establish agreement between brain regions across species, an older pig model could be used, or younger mouse and rat models. Our results suggest that the pig model used in this study is likely younger than the rodent models. Indeed, Duhaime *et al*. have suggested that a 4-month-old pig is in early adolescence^39^, indicating that a 5-month-old pig may be in adolescence instead of adulthood as is the case for the rodent models. These types of experiments should be extended to other age groups and include human samples to determine a gold standard animal model surrogate for human tissue.

## Discussion

There is a dearth of high-quality data for the viscoelastic properties of brain tissue at dynamic rates for large-strain deformations. Furthermore, differences exist in the reported properties in the literature which is possibly caused by differences in loading modalities, chosen animal model, analytical techniques, animal age, loading direction, and strain-rate^20,40^. The results presented here address this problem by conducting large-strain dynamic micro-indentations on mouse, rat, and pig brain tissue to determine viscoelastic properties which can be used to simulate the deformation behaviour of brain tissue during trauma. The effects of age, fiber direction and species on the viscoelastic properties are also investigated. High-quality, regional and age-related mechanical properties of brain tissue are essential to understand the biomechanics of traumatic brain injury and the development of preventative and surgical interventions. A full list of the means ± standard deviations of all the neo-Hookean QLV parameters for each group investigated in this study are provided in the supplementary materials.

### Age-related mechanical properties

The brain is constantly changing throughout the lifetime of an organism, from the anatomical development of the central nervous system during embryology and infant years^41,42^, to the microscale changes in protein and water content in adulthood^23^. These changes both large and small can have a marked effect on the mechanical properties of brain tissue. Here, we investigated the changes in the mechanical properties of mouse brain tissue from adolescence into early adulthood. While changes in the mean values were noted for all regions for the different age groups, only the pons region had no significant differences between age groups. For the cortex, significant differences were found between the 6–10 week-old groups and 6–12 week-old groups, but not the 10–12 week-old groups. A possible explanation for this is that only some brain regions experience significant differences in shear modulus value between the investigated age-groups when compared with the other brain regions due to marked changes in cellular composition, water and protein content occurring in the cortex when compared with other regions^23,43^. The cerebral cortex has been shown to experience more significant changes in protein, lipid, and water content with age, compared to the hippocampus in rat brains^23^. Indeed, it has been shown that the human cerebral cortex continues to change throughout life, and that grey matter density in the left posterior temporal region increases until approximately 30 years of age^44^.

The change in structure, chemistry, and mechanical properties throughout an organism’s life is relevant when investigating brain trauma using finite element models, as the mechanical properties used in these models need to be corrected for the age of the subject^45^. Furthermore, Duhaime *et al*. found a positive correlation between the percentage of hemispheric damage with increasing age for cortical injuries^39^. The current work has shown that significant differences in the mechanical properties exist for the cerebellum, cortex, and medulla oblongata between the 6 and 12-week-old murine brains. However, no significant differences were found in the pons or between the ages of 10–12 weeks for all regions. The findings presented here, that the brain’s mechanical properties change significantly with age, agree with previous results in the literature. Elkin *et al*. reported changes in the cortex modulus for P10, P17 and adult rat brains with more noticeable differences observed in hippocampal regions^23^. Furthermore, Gefen *et al*. investigated the age-dependent changes in material properties of the rat brain from postnatal days 13 (2 W), 17, 43 (6 W), and 90 (13 W) using indentation, and found no significant differences in the mechanical properties of the cerebral cortex between the 13 and 17 day-old, or the 43 and 90 day-old rats^10^. The immature cortex, however, was found to be significantly stiffer than the mature cortex in agreement with^11^ but conflicts with the results reported here and by Elkin *et al*.^23^. Our results provide an age range for which the mechanical properties of brain tissue do not change significantly (10–12 W) and could be used to provide a first-order approximation of the mechanical properties for healthy brain tissue. Future work will focus on extending this data across a wider range of age groups to determine specific age groups wherein the mechanical properties of brain tissue do not change significantly. This data would improve the accuracy and speed of injury prediction from FE models for different age groups without the need for patient-specific material properties.

### Effects of fiber direction on brain tissue mechanical properties

Evidence from anatomical, functional MRI, and DTI studies have concluded that several regions of the brain are structurally anisotropic with a few regions, such as the corpus callosum, having highly aligned fibers^21,46,47^. However, this does not necessarily translate to material anisotropic behaviour. A common misconception amongst bioengineers is that brain tissue is primarily neuronal cells with white matter being made up of highly aligned axons and grey matter consisting of the neuronal cell bodies. However, this is merely part of the picture of overall brain structure, and entire neurons are also found in cortical grey matter^42^. Furthermore, in the cerebrum, non-neuronal cells outnumber neuronal cells by almost a factor of 4^48^. But perhaps the most common misconception leading engineers to assume white matter must have material anisotropy is the textbook depiction of a neuron with a large cell body, several small dendrites and one long extending axon and axon terminal^49^. The true morphology of the neuron, however, is far more complex. Typically, a neuron will have a dendritic arbor and a terminal arbor of the axon with potentially thousands of post-synaptic specializations and presynaptic terminals, respectively^49^. The axon is located between these two arbors as a single fiber, with many connections from other axons or dendrites to be found not only at the dendritic arbors but also along the axon and between axons (axoaxonic synapse). We hypothesise that the true morphology of the axon suggests that white matter tissue is more likely to be isotropic than anisotropic. Isolated axonal tracts are likely to be anisotropic, however, as they are only one cell type from myriad components that comprise white matter tissue.

### Species-dependent viscoelastic properties of brain tissue

Human tissue is the gold standard for investigating the mechanical properties of brain tissue to study the biomechanics of TBI. However, it can be difficult to obtain human tissue that is in an appropriate condition to produce approximate *in vivo* mechanical properties, due to post-mortem time, cause of death, and age. In the absence of appropriate human tissue, animal tissue is used instead, and a porcine or rodent model is typically employed in these investigations^50^. Like human tissue, previous results in the literature have demonstrated that the mechanical properties of animal brain tissue are regionally dependent in the rat, mouse, and pig brains^13–16,21^. However, the answer as to which animal model is the most suitable surrogate remains moot. Cheng *et al*. highlighted the absence of literature concerning the direct comparison of mechanical properties of brain tissue across species^51^. Here, we address this problem by performing indentation experiments on four regions of mouse, rat, and pig brains to quantify the degree of difference between species brain regions at dynamic rates.

The neo-Hookean based viscoelastic model was fitted to the experimentally measured force-time data from indentation tests. The resulting shear moduli values for each region were compared across the three species using ANOVA with a post-hoc test. The results show that the shear modulus of the cortex is significantly different between the 10 W mouse and pig, and the rat and pig with no significant difference observed between the 10 W mouse and rat. The modulus of the pons is significantly different between the 10 W mouse and rat, but not for the mouse and pig, or rat and pig. The shear moduli of the cerebellum and medulla oblongata were insignificantly different across all three species. When the data for the 10 W mouse is replaced with the data for the 6 W mouse, no significant differences are found between the mouse and rat brains, suggesting that these may be closer in equivalent age of neural development. The cortex, however, remains significantly different between the mouse and pig, and rat and pig when using 6 W mouse data for statistical comparisons. See Fig. 5 for graph of significant differences and shear moduli values.

Previously, Pervin & Chen investigated the differences between cow, pig, and lamb brains and found no significant differences in their biomechanical response at either dynamic or quasi-static rates, although they did not account for multiple regions^19^. These results agree with many of the regions investigated in this study which indicates that the mechanical response of brain tissue across species may be surprisingly homogeneous. Prange *et al*. investigated the differences between human temporal lobe grey matter and porcine thalamic tissue^52^, and found human brain tissue to be 1.3 times stiffer than porcine tissue. However, as the results presented here and others previously reported in the literature, the mechanical properties between brain regions are significantly different and for direct comparisons to be made across species, mechanical tests should be performed in the same regions for both species. Furthermore, Nicolle *et al*. found no significant differences to exist in the linear viscoelastic properties between the porcine and human tissue measured from shear oscillatory experiments^53^. In 1970, Galford & McElhaney performed a viscoelastic study of human and monkey brain tissue and found the monkey brain to be slightly stiffer and more viscous than human brain and attributed these differences to the difference in post-mortem time effect^54^.

The previously reported results from the literature and those reported here suggest that there is significantly more homogeneity in the mechanical properties between brain regions across species than expected. Future work will involve age-matching the mechanical properties of brain tissue across different species and comparing them with those for human brain tissue. This will indicate suitable animal models that are age-matched with human brain tissue properties, allowing for selection of the most suitable surrogate to investigate the biomechanical response of brain tissue during surgery, disease, and trauma, across a range of age groups.

Several limitations must be addressed when considering the conclusions drawn from this body of work. The indented surfaces of brain tissue from all animal models were assumed to be perfectly flat, homogeneous, and perpendicular to the axis of the indenter. This was accounted for during experiments by using the digital microscope to identify regions void of vasculature and relatively flat, smooth, and perpendicular to the indenter axis. When gyri and sulci were present, indentations were always performed at the apex of each gyri. A neo-Hookean based viscoelastic framework is used here which can accurately describe the deformations generated in this work. For larger deformations than those explored here, increased non-linear behaviour may arise and a more sophisticated model, such as Ogden, may be required. However, we previously found the neo-Hookean, Mooney-Rivlin, and Ogden hyperelastic models all appear to be capable of accurately capturing the behaviour of brain tissue during indentation^13^. Furthermore, the neo-Hookean model has less parameters compared to the Mooney-Rivlin and Ogden models, and thus improves accuracy and computational time for determining the parameters of the quasi-linear viscoelastic framework. Other limitations include conducting experiments at room temperature (22 °C) and specimens were not entirely submerged in liquid during experimentation, as this could cause the tissue to swell. Instead the specimens were kept hydrated throughout experimentation by continuously applying Phosphate Buffer Solution via syringe. However, testing brain tissue at 37 °C with artificial perfusion may provide mechanical properties that are more equivalent to *in vivo* tissue and should be investigated in future work. The time-temperature superposition data generated by Hrapko *et al*.^48^ could be applied to this data and adjusted to 37 °C if required^40^. Pervin & Chen (2011) found gender to have no effect on the biomechanical response of brain tissue for cow pig and lamb brains^19^. Mixed sex mice were used in this study and it was assumed that gender had no effect on the mechanical response on mouse brain tissue, however, this was not explicitly investigated here. The effects of *in situ* boundary conditions imposed on the brain by the skull were not considered in this study. However, we have previously addressed this question in the mouse model and found no significant differences between *in situ* and *in vitro* cortex and cerebellum tissue; this is likely due to the small indenter size relative to the opening in the skull^13^.

It must also be considered that indentation is essentially a single parameter experiment and thus is not ideal for measuring anisotropic effects of tissues. Compared with other tests such as tension and shear experiments which can engage embedded fibers when deformed along the fiber direction, indentation must deform the material parallel to the fiber direction essentially loading the fibers in a three-point-bending regime, if the indenter happens to engage the fibers. A more appropriate approach to measure material anisotropy using indentation methods is to use asymmetric indentation. Feng *et al*.^55^ used this approach to characterise the anisotropic material properties of white matter under large strain and found the tissue to have a strong mechanical anisotropy^55^. However, the tests conducted by Feng *et al*.^55^ were at a rate 100 times slower and limited to small strains.

## Conclusion

This work addresses the differences in the reported mechanical properties of brain tissue by directly addressing the main biological aspects which have been shown to affect the mechanical behaviour of brain tissue: age, location, species, and fiber direction. Furthermore, we address the dearth of accurate, regional viscoelastic data for brain tissue at large-strains and dynamic strain-rates and make significant progress towards determining suitable surrogates for human brain tissue. The following conclusions can be drawn from these results:

- The mechanical properties of mouse brain tissue change during an organism’s lifetime, with significant changes occurring between 6–12 weeks in the cerebellum and cortex, and between 6–10 weeks in the medulla oblongata and cortex. No significant differences were found between any age groups for the pons.
- Mechanical properties of brain tissue are regionally dependent in all animal models.
- The corpus callosum is weakly anisotropic in pigs.
- Significant differences exist between the pons and cerebral cortex across the species investigated in this study. However, it might be possible to age match brains of different species by conducting similar experiments on an extended set of age groups.
- Regional and age-accounted mechanical properties of brain tissue across species are required for determining suitable human surrogates across the age-spectrum.

## Electronic supplementary material

Dataset

## References

[1] World Health Organization, Basso, A., Previgliano, I., Servadei, F., Neurological disorders: public health challenges, 3.10 Traumatic brain injuries, 164–176 (2006).

[2] Roozenbeek B, Maas AIR, Menon DK (2013). Changing patterns in the epidemiology of traumatic brain injury. Nature Reviews Neurology.

[3] Topolovec J (2014). Traumatic brain injury among men in an urban homeless shelter: observational study of rates and mechanisms of injury. Canadian Medical Association Journal Open.

[4] La Placa MC, Prado GR (2010). Neural mechanobiology and neuronal vulnerability to traumatic loading. J. Biomech..

[5] Greenwald RM, Gwin JT, Chu JJ, Crisco JJ (2008). Head impact severity measures for evaluating mild traumatic brain injury risk exposure. Neurosurgery.

[6] Kleiven S (2007). Predictors for traumatic brain injuries evaluated through accident reconstruction. Stapp Car Crash J..

[7] Antona-Makoshi, J., Davidsson, J., Ejima, S. & Ono, K. Reanalysis of monkey head concussion experiment data using a novel monkey finite element model to develop brain tissue injury reference values. *Proc. 2012 International IRCOBI Conference on the biomechanics of impact*. 441–454 (2012).

[8] Giordano C, Kleiven S (2014). Evaluation of axonal strain as a predictor for mild traumatic brain injuries using Finite Element modelling. Stapp Car Crash J..

[9] LaPlaca MC, Lee VM, Thibault LE (1997). An *in vitro* model of traumatic injury model to examine the response of neurons to a hydrodynamically-induced deformation. Annals of Biomedical Engineering..

[10] Gefen A, Gefen N, Zhu Q, Raghupathi R, Margulies SS (2003). Age-dependent changes in material properties of the brain and braincase of the rat. J. Neurotrauma.

[11] Prange MT, Magulies SS (2002). Regional, directional, and age-dependent properties of the brain undergoing large deformation. J. Biomech. Eng..

[12] Finan JD, Elkin BS, Pearson EM, Kalbian IL, Morrison B (2012). Viscoelastic properties of the rat brain in the sagittal plane: Effects of anatomical structure and age. Annals of Biomedical Engineering..

[13] MacManus DB, Pierrat BP, Murphy JG, Gilchrist MD (2016). Mechanical characterization of the P56 mouse brain under large deformation dynamic indentation. Sci. Rep..

[14] MacManus DB, Pierrat BP, Murphy JG, Gilchrist MD (2017). A viscoelastic analysis of the P56 mouse brain under large-deformation dynamic indentation. Acta Biomaterialia.

[15] Elkin, B. S., Ilankova, A. & Morrison B., III Dynamic, regional mechanical properties of the porcine brain: Indentation in the coronal plane. *Journal of Biomechanical Engineering*. **13**3(7), 071009-1-7 (2011). 10.1115/1.4004494 21823748

[16] Jin X, Zhu F, Mao H, Shen M, Yang KH (2013). A comprehensive experimental study on material properties of human brain tissue. J. Biomech..

[17] Rashid B, Destrade M, Gilchrist MD (2014). Mechanical characterization of brain tissue in tension at dynamic strain rates. J. Behav. Biomed. Mat..

[18] Li, K., Liu, W. & Yin, Z. Material properties and constitutive modeling of infant porcine cerebellum tissue in tension at high strain rate. *PLoS ONE*, **10**(4) (2015). 10.1371/journal.pone.0123506 PMC4382295 25830545

[19] Pervin F, Chen WW (2011). Effect of inter-species, gender, and breeding on the mechanical behavior of brain tissue. NeuroImage..

[20] Chatelin S, Constantinesco A, Willinger R (2010). Fifty years of brain tissue mechanical testing: From *in vitro* to *in vivo* investigations. Biorheology..

[21] Budday S (2017). Mechanical characterization of human brain tissue. Acta Biomaterialia..

[22] Libertiaux V, Pascon F, Cescott S (2011). Experimental verification of brain tissue incompressibility using digital image correlation. J. Mech. Behav. Biomed. Mater..

[23] Elkin, B. S., Ilankovan, A. & Morrison B. III Age-dependent regional mechanical properties of the rat hippocampus and cortex. *J Biomech Eng*. **13**2, 011010-1-10 (2010). 10.1115/1.4000164 20524748

[24] Rivlin R. S. Large elastic deformations of isotropic materials. IV. Further developments of the general theory, Philosophical Transactions of the Royal Society A, **241**(835) (1948).

[25] Puso MA, Weiss JA (1998). Finite element implementation of anisotropic quasi-linear viscoelasticity using a discrete spectrum approximation. J Biomech. Eng..

[26] Maas S., Rawlins D., Weiss J. & Ateshian, G. *FEBio Theory Manual 2.4*, Chapter 5.4 Viscoelasticity, 94–96 (2015).

[27] Ferrer I (2007). *Brain protein preservation largel*y depends on the postmortem storage temperature: Implications for study of proteins in human neurologic diseases and management of brain banks: A BrainNet Europe study. J. Neuropathol. & Exp. Neurol..

[28] Fountoulakis M, Hardmeier R, Hoger H, Lubec G (2001). Postmortem changes in the level of brain proteins. Exp. Neurol..

[29] Morisson B (2003). A tissue level tolerance criterion for living brain developed with an *in vitro* model of traumatic mechanical loading, Stapp Car Crash. Journal.

[30] Cullen DK, Simon CM, LaPlaca MC (2007). Strain-rate dependent induction of reactive astrogliosis and cell death in three-dimensional neuronal-astrocytic co-cultures. Brain Research.

[31] Budday S (2015). Mechanical properties of gray and white matter brain tissue by indentation. J. Mech. Behav. Biomed. Mater..

[32] Goriely A (2015). Mechanics of the brain: Perspectives, challenges, and opportunities. Biomech. Model. Mechanobiol..

[33] Zhang J, Green MA, Sinkus R, Bilston LE (2011). Viscoelastic properties of human cerebellum using magnetic resonance elastography. J. Biomech..

[34] Christ AF (2010). Mechanical difference between white and gray matter in the rat cerebellum measure by scanning force microscopy. J. Biomech..

[35] Pervin F, Chen WW (2009). Dynamic mechanical response of bovine gray and white matter brain tissues under compression. J. Biomech..

[36] Dutta S, Sengupta P (2016). Men and mice: relating their ages. Life Sciences..

[37] Sengupta P (2013). The laboratory rat: relating its age with human’s. Int. J. Prev. Med..

[38] Reiland S (1978). Growth and skeletal development of the pig. Acta Radiol. Suppl..

[39] Duhaime A (2000). Maturation-dependent response of the piglet brain to scaled cortical impact. J. Neurosug..

[40] Hrapko, M., van Dommelen, J. A. W., Peters, G. W. M. & Wismans, J. S. H. M. The influence of test conditions on characterization of the mechanical properties of brain tissue, *J. Biomech. Eng*. **130**, 031003-1-10 (2008). 10.1115/1.2907746 18532852

[41] Sigaard RK, Kjaer M, Pakkenberg B (2016). Development of the cell population in the brain white matter of young children. Cereb. Cortex..

[42] Budday, S., Steinmann, P. & Kuhl, E. Physical biology of human brain development. *Front. Cell. Neurosci*. **9**(257) (2015). 10.3389/fncel.2015.00257 PMC4495345 26217183

[43] Bandeira F, Lent R, Herculano-Houzel S (2009). Changing numbers of neuronal and non-neuronal cells underlie postnatal brain growth in the rat. Neuroscience..

[44] Sowell ER (2003). Mapping cortical change across the human life span. Nature Neuroscience..

[45] Antona-Makoshi J, Eliasson E, Davidsson J, Ejima S, Ono K (2015). Effect of aging on brain injury prediction in rotational head trauma – a parameter study with a rat finite element model. Traffic Inj. Prev..

[46] Pierpaoli C, Jezzard P, Basser PJ, Barnett A, Di Chiro G (1996). Diffusion tensor MR imaging of the human brain. Radiology..

[47] Riederer BM, Berbel P, Innocenti GM (2004). Neurons in the corpus callosum of the car during postnatal development. Eur. J. Neurosci..

[48] Azevedo FAC (2009). Equal numbers of neuronal and nonneuronal cells make the human brain an isometrically scaled-up primate brain. J. Comp. Neurol..

[49] Brady S. T. & Tai L. Basic Neurochemistry – *Principles of Molecular, Cellular, and Medical Neurobiology*, Chapter 1, Cell Biology of the Nervous System, 8th, (Elsevier, 2012).

[50] Xiong Y, Mahmood A, Chopp M (2013). Animal models of traumatic brain injury. Nat. Rev. Neurosci..

[51] Cheng S, Clarke EC, Bilston LE (2008). Rheological properties of the tissues of the central nervous system: A review. Medical Engineering & Physics..

[52] Prange MT, Meaney DF, Margulies SS (2000). Defining brain mechanical properties: effects of region, direction, and species. Stapp Car Crash Journal..

[53] Nicolle S, Lounis M, Willinger R (2004). Shear properties of brain tissue over a frequency range relevant for automotive impact situations: new experimental results. Stapp Car Crash Journal..

[54] Galford JE, McElhaney JH (1970). A viscoelastic study of scalp, brain, and dura. J. Biomech..

[55] Feng Y, Okamoto RJ, Namani R, Genin GM, Bayly PV (2013). Measurements of mechanical anisotropy in brain tissue and implications for transversely isotropic material models of white matter. J. Mech. Behav. Biomed. Mater..

