# Author Correction: Region and species dependent mechanical properties of adolescent and young adult brain tissue

**DOI:** 10.1038/s41598-018-28932-7

**Published:** 2018-07-20

**Authors:** David B. MacManus, Baptiste Pierrat, Jeremiah G. Murphy, Michael D. Gilchrist

**Affiliations:** 1 School of Mechanical & Materials Engineering, University College Dublin, Dublin, Ireland; 2 Ecole Nationale Supérieure des Mines de Saint-Etienne, CIS-EMSE, SAINBIOSE, F-42023 Saint Etienne, France; 3 INSERM, U1059, F-42000 Saint Etienne, France; 4 School of Mechanical & Manufacturing Engineering, Dublin City University, Dublin, Ireland

Correction to: *Scientific Reports* 10.1038/s41598-017-13727-z, published online 23 October 2017

In the original version of the Supplementary Information file published with this Article, there was a typographical error.

“μ is the shear modulus (kPa) of the neo-Hookean based viscoelastic model”

now reads:

“μ is the shear modulus (Pa) of the neo-Hookean based viscoelastic model”.

Additionally, in the original Supplementary information file, the R2 standard deviation values in the table for ‘6 week old mouse’ were omitted, the table for ‘22 week old pig’ contained errors in the rows containing the mechanical data for the cerebellum (Cbl) as well as the mean and standard deviation for the second time constant (t2) for the medulla (Md).

These errors were exclusive to the Supplementary Information and did not affect the main article. These errors have been corrected in the Supplementary Information that now accompanies the Article.

