# Supplementary material for: Region and species dependent mechanical properties of adolescent and young adult brain tissue
Source: Sci Rep. 2017 Oct 23;7:13729. doi: 10.1038/s41598-017-13727-z (PMC5653834; doi:10.1038/s41598-017-13727-z)
Supplement: Supplementary file 1 — Dataset [file 41598_2017_13727_MOESM1_ESM.pdf]

Region and species dependent mechanical properties of adolescent and young adult brain tissue

David B. MacManus[1] (david.mac-manus@ucdconnect.ie), Baptiste, Pierrat[1,2,3] (pierrat@emse.fr), Jeremiah G. Murphy[4] (jeremiah.murphy@dcu.ie), Michael D. Gilchrist[1*] (michael.gilchrist@ucd.ie)

[1]School of Mechanical & Materials Engineering, University College Dublin, Ireland

[2]Ecole Nationale Supérieure des Mines de Saint-Etienne, CIS-EMSE, SAINBIOSE, F-42023 Saint Etienne, France

[3]INSERM, U1059, F-42000 Saint Etienne, France

[4]School of Mechanical & Manufacturing Engineering, Dublin City University, Ireland

*Corresponding author: E: (michael.gilchrist@ucd.ie), T: +353 1 716 1890 F: +353 1 2830534

Cb = Cerebellum, Ctx = Cortex, Md = Medulla oblongata, Po = Pons, Th = Thalamus (sagittal plane), CRC = Corona Radiata (coronal plane), CCC = Corpus callosum (coronal plane), CCS = Corpus callosum (sagittal plane). μ is the shear modulus (Pa) of the neo-Hookean based viscoelastic model, g1, g2, and g∞ are the reduced relaxation functions, t1 and t2 are the time constants in seconds.

	**6 week old mouse**							
		**μ**	**g1**	**t1**	**g2**	**t2**	**g∞**	**R2**
**Cbl**	**MEAN**	2475	0.492	0.021	0.221	0.304	0.287	0.977
	**STD**	390	0.014	0.000	0.007	0.007	0.013	0.011
**Ctx**	**MEAN**	4831	0.520	0.023	0.218	0.303	0.262	0.978
	**STD**	1012	0.021	0.004	0.023	0.009	0.017	0.008
**Md**	**MEAN**	3813	0.553	0.020	0.221	0.278	0.225	0.977
	**STD**	860	0.036	0.001	0.015	0.003	0.029	0.013
**Po**	**MEAN**	5656	0.593	0.022	0.209	0.278	0.198	0.980
	**STD**	1092	0.025	0.002	0.010	0.003	0.023	0.013

	10 weeks old mouse							
		µ	g1	t1	g2	t2	g∞	R2
Cbl	MEAN	2807	0.536	0.022	0.209	0.328	0.255	0.967
	STD	544	0.026	0.002	0.010	0.028	0.030	0.011
Ctx	MEAN	6752	0.555	0.024	0.206	0.353	0.240	0.963
	STD	1736	0.024	0.001	0.011	0.004	0.023	0.036
Md	MEAN	4651	0.584	0.024	0.200	0.353	0.216	0.963
	STD	714	0.022	0.001	0.011	0.003	0.019	0.013
Po	MEAN	6564	0.599	0.021	0.206	0.351	0.196	0.970
	STD	1690	0.012	0.001	0.013	0.020	0.014	0.009

	12 weeks old mouse							
		µ	g1	t1	g2	t2	g∞	R2
Cbl	MEAN	3142	0.508	0.025	0.203	0.299	0.290	0.982
	STD	1082	0.019	0.002	0.017	0.004	0.020	0.004
Ctx	MEAN	7668	0.553	0.026	0.202	0.396	0.246	0.981
	STD	478	0.012	0.004	0.003	0.032	0.010	0.005
Md	MEAN	4317	0.571	0.022	0.203	0.283	0.227	0.983
	STD	566	0.017	0.000	0.006	0.002	0.015	0.003
Po	MEAN	6509	0.621	0.018	0.201	0.284	0.178	0.980
	STD	1514	0.032	0.001	0.019	0.003	0.025	0.010

	20-25 week old rat							
		μ	g1	t1	g2	t2	g_∞	R2
**Cbl**	**MEAN**	2611	0.515	0.020	0.187	0.302	0.298	0.971
	**STD**	497	0.031	0.001	0.016	0.003	0.021	0.009
**Ctx**	**MEAN**	5715	0.534	0.020	0.207	0.304	0.258	0.980
	**STD**	2089	0.030	0.001	0.013	0.011	0.021	0.007
**Md**	**MEAN**	4602	0.620	0.019	0.176	0.280	0.204	0.974
	**STD**	1736	0.036	0.002	0.016	0.034	0.027	0.010
**Po**	**MEAN**	4933	0.643	0.018	0.173	0.299	0.184	0.977
	**STD**	1005	0.034	0.002	0.016	0.016	0.026	0.007

	22 week old pig							
		μ	g1	t1	g2	t2	g_∞	R2
**Cbl**	**MEAN**	2827	0.504	0.017	0.169	0.302	0.327	0.959
	**STD**	631	0.032	0.001	0.015	0.002	0.039	0.012
**Ctx**	**MEAN**	3200	0.496	0.019	0.167	0.295	0.337	0.953
	**STD**	565	0.041	0.001	0.023	0.004	0.059	0.023
**Md**	**MEAN**	5307	0.532	0.018	0.201	0.246	0.267	0.979
	**STD**	1673	0.031	0.001	0.006	0.025	0.032	0.007
**Po**	**MEAN**	6192	0.518	0.017	0.196	0.263	0.286	0.977
	**STD**	2235	0.062	0.002	0.009	0.007	0.068	0.010
**Th**	**MEAN**	2806	0.524	0.021	0.200	0.281	0.276	0.969
	**STD**	288	0.028	0.003	0.012	0.005	0.025	0.014
**CR**	**MEAN**	5623	0.634	0.017	0.187	0.262	0.179	0.975
	**STD**	988	0.028	0.001	0.013	0.002	0.020	0.009
**CCC**	**MEAN**	5344	0.611	0.021	0.196	0.283	0.193	0.977
	**STD**	1358	0.027	0.001	0.008	0.003	0.023	0.006

**CCS**	**MEAN**	4894	0.560	0.018	0.189	0.273	0.251	0.969
	**STD**	983	0.051	0.002	0.009	0.010	0.052	0.007

Experimental parameters, number of indentations per region and age group

Age (weeks)	Cerebellum	Cortex	Medulla	Pons
**6**	15	15	16	23
**10**	30	30	30	30
**12**	10	10	11	11
